# HIV Incidence Prior to, during, and after Violent Conflict in 36 Sub-Saharan African Nations, 1990-2012: An Ecological Study

**DOI:** 10.1371/journal.pone.0142343

**Published:** 2015-11-12

**Authors:** Brady W. Bennett, Brandon D. L. Marshall, Annie Gjelsvik, Stephen T. McGarvey, Mark N. Lurie

**Affiliations:** Department of Epidemiology, Brown University School of Public Health, Providence, RI, United States of America; British Columbia Centre for Excellence in HIV/AIDS, CANADA

## Abstract

**Objectives:**

The aim of this study was to determine the association between violent conflict and HIV incidence within and across 36 sub-Saharan Africa countries between 1990 and 2012.

**Methods:**

We used generalized linear mixed effect modeling to estimate the effect of conflict periods on country-level HIV incidence. We specified random intercepts and slopes to account for across and within country variation over time. We also conducted a sub-analysis of countries who experienced conflict to assess the effect of conflict intensity on country-level HIV incidence. All models controlled for level of economic development, number of refugees present in the country, and year.

**Results:**

We found that, compared to times of peace, the HIV incidence rate increased by 2.1 per 1000 infections per year (95%CI: 0.39, 3.87) in the 5 years prior to conflict. Additionally, we found a decrease of 0.7 new infections per 1000 people per year (95%CI: -1.44, -0.01) in conflicts with 25 to 1000 battle-related deaths and a decrease of 1.5 new infections per 1000 people per year (95%CI:-2.50, -0.52) for conflict with more than 1000 battle-related deaths, compared to conflicts with less than 25 battle-related deaths

**Conclusions:**

Our results demonstrate that HIV infection rates increase in the years immediately prior to times of conflict; however, we did not identify a significant increase during and immediately following periods of violent conflict. Further investigation, including more rigorous data collection, is needed, as is increased aid to nations at risk of violent conflict to help in the fight against HIV/AIDS in sub-Saharan Africa.

## Introduction

Since its discovery over three decades ago, HIV/AIDS has caused nearly 40 million deaths [1] with the overwhelming majority occurring in sub-Saharan Africa. Simultaneously, sub-Saharan Africa has been ravaged by decade long wars in several countries, leading to a dual burden of violent conflict and infectious disease [2]. Some studies have shown that conflicts help propagate the spread of HIV/AIDS leading to higher HIV incidence rates during times of violent conflict [3–4]. However, several recent publications have challenged this proposition with results suggesting the opposing hypothesis that HIV infection rates stabilize or decrease during conflict [5–7]. Further research is necessary to fully understand the association between conflict and HIV incidence, so that the global health community can be better prepared to meet the needs of areas dually affected by HIV/AIDS and violent conflict.

### Conflict as a Propagative Agent

Numerous factors related to violent conflict are known to contribute to the spread of HIV. Most notable among these are sexual violence [8–9], the vast movements of refugees and/or internally displaced peoples (IDPs) [5, 10–11], the higher than average prevalence of HIV/AIDS among men in the military [3,11], and disrupted access to health care services [7]. Additionally, rape and sexual violence places women and children at particularly high risk of HIV/AIDS and other sexually transmitted infections (STIs) [9,12]. A recent report of seven conflict affected nations in sub-Saharan Africa showed that in countries with conflicts characterized by widespread sexual violence, annual HIV incidence increased by up to 7% [8]. Another study among households surveyed in three IDP camps in Sierra Leone showed that 13% reported incidents of war-related abuses with 9% of all respondents and 8% of women reporting sexual violence [13]. The Joint United Nations Program on HIV/AIDS (UNAIDS) has documented that the Democratic Republic of the Congo, Uganda, Liberia, and Rwanda have all experienced conflicts involving rape as a weapon of war [14].

It has also been reported that individuals who are deprived of their normal social and economic networks, particularly those displaced for extended periods of time, are more likely to engage in high-risk behaviors, thus increasing their vulnerability to HIV infection [5,10,12–13]. However, this finding has not been uniformly observed across nations [6]. A 2007 systematic review examined the HIV prevalence among refugee populations compared to host communities and found no significant increase among seven nations that experienced prolonged armed conflict. While several of the refugee camps experienced a decrease in HIV prevalence when comparing the host country to the refugee camp, Burundian camps in Tanzania and Eritrean camps in Sudan reported either an increase or unchanged HIV prevalence [6]. However, these studies failed to compare groups of people with similar baseline HIV prevalence rates; therefore, one cannot concretely say whether HIV infections increased or decreased in these refugee camps compared to non-refugee populations. These studies further illuminate the discrepancy found in HIV data collection and the necessary caution that must be taken when interpreting results from different locations during and immediately following conflict.

Another characteristic of violent conflict that potentially increases the spread of HIV/AIDS is the destruction and collapse of health infrastructure. Studies have shown that healthcare spending is one of the first government expenditures to decline in combat zones [15]. Decreases in spending and the collapse of health systems infrastructure can lead to the absence of HIV/AIDS treatment and thus inadequate suppression, resulting in increased transmission during unprotected sexual contact. For example, during Angola’s nearly 30 year war ending in 2002, all healthcare infrastructure outside of the capital city, Luanda, collapsed, and following the war the country experienced an unexpectedly high prevalence of HIV/AIDS in antenatal clinics compared to before and during the conflict [16–18].

### Conflict as a Protective Agent

The characteristics of violent conflict are not always associated with potentially increased risk of HIV/AIDS transmission. HIV prevalence measurements taken during times of war in Angola, Mozambique, Somalia, South Sudan, and Sierra Leone show a decrease of HIV infection rates during war [6,17]. Conflict sometimes has the effect of isolating communities by destroying viable transportation systems (roads, bridges, etc.) and making travel more dangerous than in times of peace [7]. Additionally, the disruption of some sexual activity (e.g. visiting sex workers, having multiple sexual partners), and the influx of humanitarian aid may slow the spread of HIV/AIDS and depress prevalence and incidence [5].

**Fig 1:**
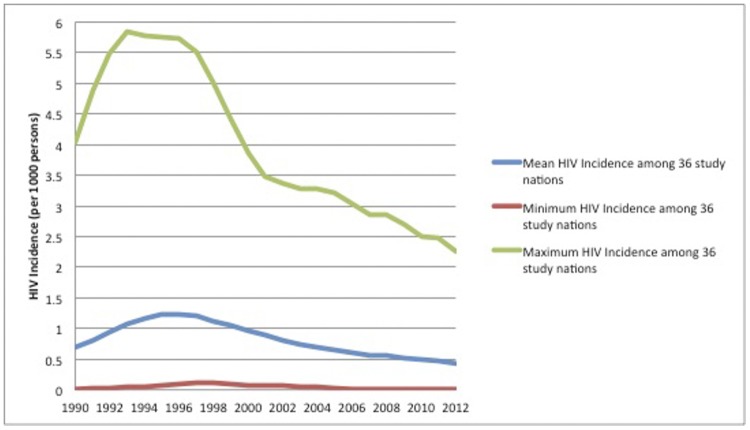
Annual Minimum, Maximum, and Mean HIV Incidence in 36 Countries in sub-Saharan Africa, 1990–2012. Adapted from UNAIDS dataset, 2014 [21].

More recent research suggests that the time period immediately following the end of a conflict could be most highly associated with transmission of HIV [16]. As transportation resumes, populations with previously low HIV prevalence can move from rural villages to urban centers, allowing many of the formerly separated populations to mix again with those of higher HIV prevalence [19–20]. The most pronounced example of this is found following Mozambique’s lengthy period of conflict and unrest (1964–1992). During conflict, HIV prevalence rates were less than 1% [16]; However, in the 12 years following a peace settlement, the prevalence of HIV among high risk populations such as pregnant women rose dramatically to an average of greater than 20% by 2004 [21]. However, nearly all countries in sub-Saharan Africa had low prevalence of HIV/AIDS prior to 1992 (Fig 1) and the overall prevalence increased in all nations regardless of the presence of war through the beginning of the 21^st^ century [1].

#### Need for Empirical Studies

While numerous articles discuss the complex relationship between HIV/AIDS and violent conflict, few empirical analyses exist. The only large-scale, quantitative analysis to date was completed in 2010 and related domestic and international conflict to HIV prevalence, however, the researchers did not examine the association between HIV and the presence of violent conflict; moreover, the study suffered from numerous methodological challenges arising from the limitations of the data itself [22]. Nations in conflict often lack reliable or consistent testing for infectious diseases, and other sources of data collection (for example, that collected by humanitarian or non-governmental organizations) sometimes cease during times of conflict given high levels of violence and political instability. In addition, many studies also rely solely on HIV prevalence data, which becomes an unreliable proxy measure for HIV incidence when considering the high rate of death related to both the conflict and the disease.

Another consideration is the rise in HIV/AIDS rates for nations which have remained relatively peaceful. Many southern African nations like Zimbabwe, Lesotho, Swaziland, and Botswana did not experience more than one year of violent conflict from 1990–2012 [21]; however, each of their HIV/AIDS prevalence rates among adults reached upwards of 20% during this period [21]. In contrast, many of the nations who did experience prolonged violent conflict from 1990–2012 had markedly lower HIV prevalence rates. Among these nations, Liberia, Burundi, Angola, and the Democratic Republic of the Congo, which each experienced multiple conflicts or one prolonged conflict that encompassed several years, had the highest HIV prevalence rates with the largest recorded in Burundi in 1995–1996 at 4.5% among adults [21,23].

The proliferation of violent conflict in sub-Saharan Africa combined with the continued high HIV/AIDS infections rates require better understandings of the complexities of this relationship. The primary aims of this paper are: 1) to quantify the association between violent conflict and HIV/AIDS incidence rates in 36 sub-Saharan African nations from 1990 to 2012, and 2) to determine the temporal patterns of HIV incidence in relation to violent conflict (pre, during, and post conflict). These considerations will allow for a more complete understanding of the dynamics of HIV/AIDS in sub-Saharan Africa, informing public health professionals, clinicians, and governments about how best to prepare for HIV/AIDS before, during or after conflict.

## Methods

### Data Collection and Definitions

Data were compiled from publicly available databases. Adult HIV/AIDS incidence data by country from 1990–2012 were collected from UNAIDS [21], which compiles various sources of data (primarily HIV prevalence rates) to support mathematically predicted incidence rates through the Spectrum software. Conflict related data was retrieved from the Uppsala Conflict Data Program/Peace Research Institute Oslo (UCDP/PRIO) armed conflict dataset [22]. UCDP compiles the approximate dates of conflicts, aggravating parties, number of battle-related deaths, and other variables that define the timing and scale of a violent conflict.

Data was compiled for a total of 36 sub-Saharan African nations that had both UNAIDS HIV incidence data and UCDP conflict data for the time period 1990–2012.

Our primary outcome variable was HIV incidence, defined as the number of new infections per 1000 persons per year among adults aged 15–49 years. Our primary independent variables were the time period of conflict variable, which was defined as No Conflict, Pre-Conflict, During Conflict, and Post-Conflict, and conflict intensity, which was operationalized as a categorical variable from the battle-related deaths count (<25 battle-related deaths, 25–1000 battle-related deaths, 1000 battle-related deaths) [23,24]. Pre-Conflict was defined as five years prior to a conflict occurring, while Post-Conflict was five years after cessation. No Conflict was all times that fell outside of Pre-Conflict, During Conflict, and Post-Conflict. Other variables that were considered for inclusion in the analysis were those that could potentially confound the association between conflict and HIV incidence, such as refugee and internally displaced people count (retrieved from UNHCR for years 2000–2012) [25], conflict type (extrasystemic armed conflict, interstate armed conflict, internal armed conflict, internationalized internal conflict) [26], and level of development as defined by the World Bank based upon annual GNI per capita in constant $US, Atlas method [27], and an ordinal variable numbered 1–23 representing the year of conflict. Details of each variable definition and distribution are provided in Table 1. The data was collected and examined at the national level rather than at the individual level and used to predict national level means, therefore, the mean and median values are not weighted

**Table 1:** Definitions and Range of Dependent and Independent Variables.

Variable	Explanation	Source	Mean (95%CI)	N (%)
HIV/AIDS Incidence	Average number of new cases of HIV/AIDS over susceptible persons per year among adults, ages 15–49 (1990–2012)	UNAIDS	0.84 (0.77, 0.92)	----------------
Conflict Time Period		UCDP/PRIO	----------------	
None	No Conflict			144 (17.39%)
Pre-Conflict	Up to 5 years prior to conflict			198 (23.91%)
During Conflict	Years during conflict			68 (8.21%)
Post-Conflict	Up to 5 years following conflict			418 (50.48%)
Refugee Number	Number of refugees from nation in conflict (in thousands)	UNHCR	82.55 (71.22, 93.88)	----------------
GNI per Capita, Atlas Method	Gross National Income per capita in constant US dollars	World Bank	953.97 (914.48, 1402.56)	----------------
Conflict Intensity	1. <25 Battle Deaths	UCDP/PRIO	----------------	305 (60.28%
	2. 25–100 Battle Deaths			154 (30.43%)
	3. >1000 Battle Deaths			47 (9.29%)
Conflict Type	1. Extrasystemic	UCDP/PRIO	----------------	0 (0.00%)
	2. Interstate			5 (2.44%)
	3. Internal			150 (73.17%)
	4. International War			50 (24.39%)
Battle-Related Deaths	Number of deaths related to the violent conflict	UCDP/PRIO	1494.10 (665.01, 2323.20)	----------------

*Mean across 36 selected nations, 1990–2012

#### Statistical Methods

Descriptive statistics were used to portray HIV incidence per 1000 persons across the conflict time period categories for each of the 36 nations included in the study (Table 2).

**Table 2:** Years of Conflict and Mean HIV Incidence (number of new cases per 1000 persons per year) by Conflict Period for 36 Nations in sub-Saharan Africa, 1990–2012.

Country	Year(s) of Conflict	HIV Incidence			
		Non-Conflict	Pre-Conflict	During Conflict	Post-Conflict
Angola	1990–1995, 1998–2002, 2004, 2007, 2009	---	---	0.2357	0.26
Botswana	No Conflict 1990–2012	3.1565	---	---	---
Burkina Faso	No Conflict 1990–2012	0.145	---	---	0.225
Burundi	1991–1992, 1994–2006, 2008	---	0.61	0.314	0.225
Cameroon	1996	0.4458	0.586	0.9	0.804
Central African Republic	2001–2002, 2006, 2009–2012	1.2633	1.2007	0.4092	0.3792
Chad	1990–1994, 1997–2003, 2005–2010	---	---	0.4111	0.386
Congo	1993, 1997–1999, 2002	0.21	0.82	0.58	0.465
Cote d'Ivoire	2002–2004, 2011	0.8857	0.7383	0.355	0.2167
Democratic Republic of Congo	1996–2001, 2006–2008, 2012	0.16	0.174	0.145	0.1171
Djibouti	1991–1994, 1999, 2008		0.1156	0.1683	0.2569
Eritrea	1997–2000, 2003	0.1317	0.41	0.182	0.0371
Ethiopia	1990–1994, 1996, 1998–2012	---	---	0.2659	0.51
Gabon	No Conflict 1990–2012	0.3474	---	---	---
Gambia, The	No Conflict 1990–2012	0.1304	---	---	---
Ghana	No Conflict 1990–2012	0.2178	---	---	---
Guinea-Bissau	1998–1999	0.3309	0.282	0.495	0.556
Kenya	No Conflict 1990–2012	0.947	---	---	---
Lesotho	1998	2.1867	4.114	4.72	3.398
Liberia	1990, 2000–2003	0.01	0.37	0.224	0.152
Malawi	No Conflict 1990–2012	1.6943	---	---	---
Mali	1990, 1994, 2007–2009, 2012	0.16	0.108	0.1183	0.204
Mozambique	1990–1992	1.4453	---	0.3833	0.926
Namibia	No Conflict 1990–2012	1.5483	---	---	---
Niger	1990–1992, 1994–1995, 1997, 2007–2008	---	0.06	0.0838	0.0918
Nigeria	2004, 2011–2012	0.3856	0.53	0.305	0.34
Rwanda	1990–1994, 1996–2002, 2009–2012	---	0.18	0.4694	0.27
Sierra Leone	1991–2001	0.1217	0.02	0.1155	0.248
Somalia	1990–1996, 2001–2002, 2006–2012	---	---	0.0696	0.0843
South Africa	No Conflict 1990–2012	2.0684	---	---	0.4825
Swaziland	No Conflict 1990–2012	3.187	---	---	---
Togo	No Conflict 1990–2012	0.4519	---	---	0.305
Uganda	1994–2011	---	1.815	0.7056	0.77
United Republic of Tanzania	No Conflict 1990–2012	0.73	---	---	---
Zambia	No Conflict 1990–2012	1.7509	---	---	---
Zimbabwe	No Conflict 1990–2012	2.86	---	---	---

”---”represents data that is not applicable to that category. For example, Zimbabwe did not experience conflict 1990–2012 and therefore did not have a mean HIV incidence for Pre, During, or Post-Conflict periods.

ANOVA was used to test categorical variables for differences in the mean HIV incidence in categorical variables (Table 3). A Spearman correlation coefficient was used to compare continuous variables. Simple linear regression analyses were then used with each individual independent variable included in the model to assess for confounding. Variables that did not have a significant relationship (p<0.05) with the outcome were not included in the final model. The number of internally displaced peoples was considered along with the number of refugees as a key variable to account for the movement of people during a conflict. However, the number of IDPs did not have a significant association with HIV incidence in simple linear regression models and data could only be gathered for years 2000–2012; therefore, it was excluded from the model. The number of refugees, though not significant in simple linear regression was included in final models since previously published models include refugees as a covariate

**Table 3:** Descriptive Statistics of HIV Incidence (number of new cases per 1000 persons per year) and Covariates, 1990–2012 for 36 countries in sub-Saharan Africa.

	Mean	SD	ANOVA(Prob>F)
**Conflict Time Period**			
Non-Conflict	1.26	0.06	
Pre	0.80	0.13	<0.001
During	0.33	0.03	
Post	0.40	0.05	
**Conflict Intensity**			
<25 Battle-Related Deaths	1.00	0.05	
25–1000 Battle-Related Deaths	0.38	0.04	<0.001
>1000 Battle-Related Deaths	0.29	0.03	
**Level Development**			
Low-income	0.69	0.04	
Lower-Middle	1.79	0.14	<0.001
Upper-Middle/Upper	0.97	0.10	
		**Spearman**	**Prob>|r|**
**Number of Refugees**		0.01	0.70

To determine the independent relationship between violent conflict and adult HIV incidence and to account for the difference in adult HIV incidence within and across countries over time, a linear mixed effects regression model with random intercepts and slopes was constructed [28]. In primary analyses, the categorical conflict period variable was considered to be the primary predictor. In a sub-analysis restricted to countries that experienced at least one conflict during the period, we examined the effect of conflict intensity on HIV incidence. All statistical analyses were performed using SAS v.9.4.

## Results

Conflict was highly prevalent during the time period 1990–2012. Only 14 of the 36 nations (38.9%) experienced no conflict for the entire 23-year period. Of those 14, three had completed a conflict in the 5 years prior to the beginning of our study period (Burkina Faso, South Africa, and Togo). The 36 countries contributed 828 total observations of annual HIV incidence over the study period. Of those 828 observations, 409 (49.4%) were considered non-conflict, 68 (8.2%) “pre-conflict”, 198 (23.9%) “during conflict”, and 153 (18.5%) were characterized as “post-conflict”. Conflict intensity, which primarily shows the distribution of conflict, had 630 (76.1%) observations with <25 battle-related deaths, 148 (17.9%) with 25–1000 battle-related deaths, and 50 (6.0%) with >1000 battle-related deaths.

Over the study period, HIV incidence in sub-Saharan Africa declined overall (Fig 1). HIV incidence peaked in 1996 in sub-Saharan Africa at 12.4 new infections per 1000 people per year on average per country, with the highest recorded incidence in Zimbabwe at 58.4 new infections per 1000 people in 1993. The lowest recorded HIV incidence was experienced in 3 different nations: Eritrea 2006–2008, Liberia 2008–2012, and Niger 2008–2012; all had recorded HIV incidence estimates of 0.01 new infections per 1000 people per year.

### Model 1

The mixed effect model demonstrated several significant relationships between HIV incidence and included covariates. Pre-Conflict was associated with an increase of 2.1 new infections per 1000 persons per year (95% CI: 0.39, 3.87) compared to times of non-conflict after adjustment for year, level of development, and number of refugees and accounting for between and within-country differences (see Table 4). However, HIV incidence was not significantly increased during conflict periods (0.07 new infections per 1000 persons per year, 95% CI: -1.66, 1.79) or immediately after conflict (1.1 new infections per 1000 persons per year, 95%CI: -0.45, 2.69) compared to periods of non-conflict in multivariate analysis. The economic level of development was associated with a significant increase in HIV incidence. Specifically, compared to upper-middle/upper income countries, both low-income nations and lower middle-income countries (LMICs) showed significantly positive associations with HIV incidence. Low income nations estimated an increase of 8.0 new infections per 1000 people in HIV incidence on average per year (95%CI: 5.19, 10.86) while LMIC’s estimated an increase of 7.3 new infections per 1000 people per year (95%CI: 4.96, 9.56). Year was negatively associated with HIV incidence. For each year increase there was an average decrease of 0.3 new infections per 1000 people per year in HIV incidence (95%CI: -0.45, -0.09), illustrating the general decrease in HIV incidence seen over the past two decades.

**Table 4:** Mixed Effect Model of HIV Incidence (number of new cases per 1000 persons per year) across Conflict with a Country Specific Random Intercept and Slope in 36 Countries in sub-Saharan Africa, 1990–2012.

	Model 1^a^	Model 2^b^
	Estimate (95%CI)	Estimate (95%CI)
Intercept	3.87 (-1.60, 9.02)	4.88 (0.56, 9.17)
Conflict Period		----------------
Non-Conflict	REF	
Pre-Conflit	2.14 (0.39, 3.88)	
During Conflict	0.07 (-1.66, 1.77)	
Post Conflict	1.12 (-0.45, 2.69)	
Conflict Intensity	----------------	
No Conflict		REF
<25 Battle-Related Deaths		0.12 (-1.11, 1.32)
25–1000 Battle-Related Deaths		-0.70 (-1.44, -0.01)
>1000 Battle-Related Deaths		-1.51 (-2.50, -0.52)
Level of Development		
Low-Income	8.02 (5.19, 10.86)	1.82 (-1.67, 5.29)
Lower-Middle	7.26 (4.96, 9.56)	0.18 (-3.55, 3.16)
Upper-Middle/Upper	REF	REF
Number of Refugees	-0.002 (-0.01, 0.01)	-3.00E-04 (-0.01, 0.01)
Year	-0.27 (-0.45, -0.09)	-0.11 (-0.23, 0.01)

^a^Among all 36 nations

^b^Among 24 nations who experienced at least 1 year of conflict

### Model 2

The second model, which was limited to nations that experienced at least one year of conflict during the study period (Table 4), indicates an inverse relationship between HIV incidence and conflict intensity measured by the number of battle-related deaths. For conflicts with a battle-related death toll between 25 and 1000, HIV incidence decreased on average by 0.7 new infections per 1000 people per year (95%CI: -1.44, -0.01) and by 1.5 new infections per 1000 people per year (95%CI: -2.50, -0.52)for conflicts with >1000 battle-related deaths compared to times of non-conflict (<25 battle-related deaths). In contrast to the first model, measurements of level of development are no longer significant in Model 2, while year is associated with a marginally significant effect.

## Discussion

Our results demonstrate that, across 36 nations in sub-Saharan Africa from 1990–2012, HIV incidence was significantly higher in the five years prior to a violent conflict compared to times of non-conflict when accounting for the level of development, the number of refugees, and time in years. Furthermore, our findings indicate that the level of conflict intensity (as measured by the number of battle-related deaths) is inversely related to HIV incidence. Given that mixed effects regression models account for country-level differences and changes over time, these findings present robust evidence regarding the association between HIV/AIDS infection rates and violent conflict. Our research also supports past findings that HIV infection rates stabilized during times of conflict [16–18]. Some have noted that the time immediately following a conflict may be the most dangerous period for widespread HIV infection [6,19]. While our findings did not discover a significant association between HIV incidence and the post-conflict period, more thorough research into at-risk populations (women, commercial sex workers) must be completed to draw more concrete conclusions.

It is also important to note the significant inverse relationship between conflict intensity and HIV incidence found in the second mixed effect regression model. Several explanations exist for this finding and must be considered when making inferences about the overall effect conflict has on HIV infection rates. As conflict intensity increases as a function of battle-related deaths, there is likely increased difficulty in adequate HIV testing. Additionally, it is difficult to accurately account for the number of battle related deaths and thus conflict intensity in data collection. Our findings regarding the association of conflict intensity and HIV infections rates concur with previous studies [18], highlighting the need for stronger data collection of conflict-associated variables. Furthermore, our results build upon both previous theories that violent conflict is associated with an increase in HIV infection rates and with a plateau in HIV infection rates. Our finding in the multivariate model that HIV incidence remains stable during conflict supports the theory that conflict does not necessarily cause an increase in HIV infection despite numerous risk factors (e.g.sexual violence, interruption of medical care). However, our finding that the 5 years prior to violent conflict is associated with a significant increase in HIV incidence also support the theory that HIV infection rates are increased in the conflict setting. Further research into the socio-political environment leading up to conflict is needed to understand the intricacies of the pre-conflict time period and its association with HIV.

This study contains several limitations that are important to note. First, we used an ecological design to model the association between violent conflict and HIV incidence. While this allows us to observe nationwide trends in HIV incidence, lower national incidence rates may mask sub-epidemics within the country, and results cannot be generalized to an individual’s risk for HIV in the context of conflict. Furthermore, any study of trans-national HIV infection rates is subject to measurement errors. While UNAIDS uses Spectrum, a complex compilation of serial prevalence surveys and mathematical modeling to estimate HIV incidence [29], there is opportunity for error in measuring and predicting the true HIV incidence [29–30]. These difficulties are likely more apparent during times of conflict when testing and reporting are unreliable, and when the underlying population size may change as a result of displacement and migration in ways that are difficult to measure. Therefore, conclusions drawn from nations long at war, such as Democratic Republic of the Congo, must be taken with caution. Also, this study does not take into account the specific geographies of conflict and HIV incidence. Another limitation is the exclusion of antiretroviral scale-up measures as a covariate. The invention of highly active antiretroviral therapy (HAART) in 1996 and the scale-up of treatment in sub-Saharan Africa will both effect the overall trend of HIV incidence and, in the case of violent conflict, the after effect of HAART disruption during conflict. Recent studies have indicated that HAART disruption during violent conflict can lead to virologic resistance and increased morbidity [31–32]. This paper did not analyze data at the regional or community level, which would add a level of robustness. Future analyses will examine the effects of conflict on HIV incidence in multi-level analyses using both regional and country-level data. Future studies should also consider the community-level recording of conflict and compare HIV incidence between conflict-affected communities within a country compared to unaffected communities within that same nation. Finally, we were unable to measure the effect of behaviors that place certain groups at increased risk of HIV infection. As this was an ecological study, we had no way of collecting individual measurements of activities such as sexual behavior or sexual violence.

Strengths of this study include the use of linear mixed effect regression modeling to account for both fixed and random effects across the study nations and adjust for potentially confounding factors like number of refugees and the socioeconomic level of development. Furthermore, by accounting for both a random intercept (country) and a random slope (year), we were able to model the change in HIV incidence both within and across countries over the 23-year study period.

These results are important for public health professionals within sub-Saharan African nations and also for policymakers and stakeholders. The years preceding a conflict may represent a critical period during which clinicians and public health professionals should be most active in HIV testing, treatment provision, education, and advocacy. In order for adequate testing and education to occur, there must be cooperation from domestic policy makers and foreign nations providing aid to the people of the warring nation [33].

## Conclusion

Accounting for differences in changes and patterns in HIV incidence across nations and adjusting for other key historical and political-economic factors, the pre-conflict period has a positive association with new HIV infections in sub-Saharan Africa compared to non-conflict periods. This study represents a call for further investigation and increased aid to facing violent conflict to stave off and diminish the effects of HIV spread.

## Supporting Information

S1 Fig Average HIV Incidence in 36 sub-Saharan African Nations Across Conflict Periods. HIV Incidence data from UNAIDS dataset, 2014 [21].
(PDF)
