# Supplementary figures and images for: HIV Incidence Prior to, during, and after Violent Conflict in 36 Sub-Saharan African Nations, 1990-2012: An Ecological Study
Source: PLoS One. 2015 Nov 12;10(11):e0142343. doi: 10.1371/journal.pone.0142343 (PMC4642881; doi:10.1371/journal.pone.0142343)

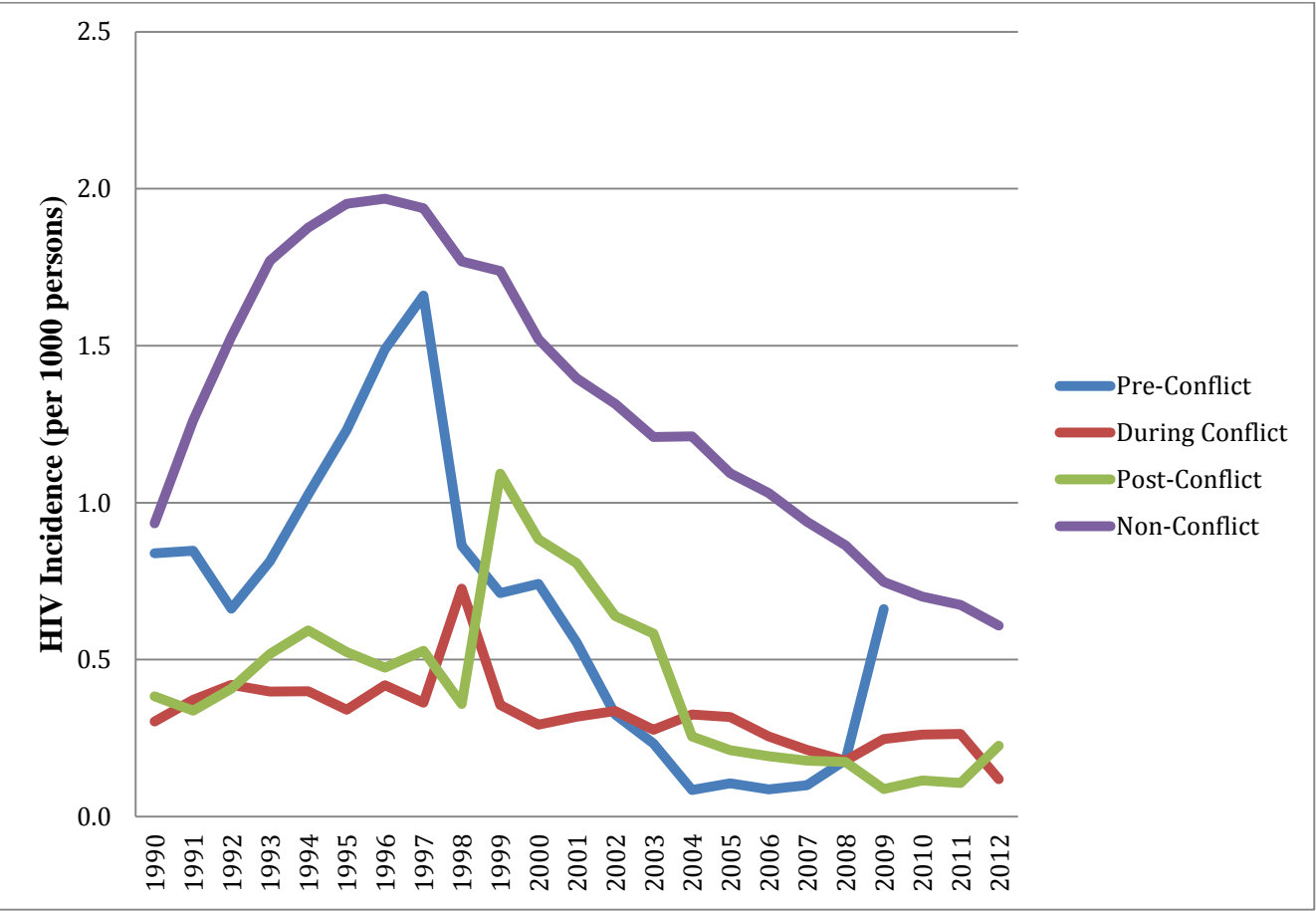

Supplement: S1 Fig — HIV Incidence data from UNAIDS dataset, 2014 [21]. (PDF) [file pone.0142343.s001.pdf]
